# Genetic markers associated with host status and clonal expansion of Group B Streptococcus in the Netherlands

**DOI:** 10.3389/fmicb.2024.1410651

**Published:** 2024-07-10

**Authors:** Uzma Basit Khan, Victoria Dyster, Chrispin Chaguza, Nina M. van Sorge, Diederik van de Beek, Wing Kit Man, Stephen D. Bentley, Merijn W. Bijlsma, Dorota Jamrozy

**Affiliations:** ^1^Parasites and Microbes Programme, Wellcome Sanger Institute, Wellcome Genome Campus, Hinxton, United Kingdom; ^2^Department of Epidemiology of Microbial Diseases, Yale School of Public Health, Yale University, New Haven, CT, United States; ^3^Department of Medical Microbiology and Infection Prevention, Amsterdam Infection and Immunity, Amsterdam UMC, University of Amsterdam, Amsterdam, Netherlands; ^4^Netherlands Reference Laboratory for Bacterial Meningitis, Amsterdam UMC Location AMC, Amsterdam, Netherlands; ^5^Department of Neurology, Amsterdam Neuroscience, Amsterdam UMC, University of Amsterdam, Amsterdam, Netherlands; ^6^Department of Paediatrics, Amsterdam UMC, University of Amsterdam, Amsterdam, Netherlands

**Keywords:** Group B Streptococcus, neonatal invasive disease, maternal carriage, serotypes, clonal complexes, mobile genetic elements

## Abstract

**Objectives:**

Certain Group B Streptococcus (GBS) genotypes are associated with invasive disease in neonates. We conducted a comparative genomic analysis of GBS isolates from neonatal disease and maternal carriage in the Netherlands to determine distribution of genetic markers between the two host groups.

**Methods:**

Whole genome sequencing was used to characterise 685 neonatal invasive isolates (2006–2021) and 733 maternal carriage isolates (2017–2021) collected in the Netherlands.

**Results:**

Clonal complex (CC) 17 and serotype III were significantly more common in disease while carriage isolates were associated with serotypes II, IV, V as well as CC1. Previously reported CC17-A1 sub-lineage was dominant among disease isolates and significantly less common in carriage. The phiStag1 phage, previously associated with expansion of invasive CC17 isolates in the Netherlands, was more common among disease isolates compared to carriage isolates overall, however it was equally distributed between CC17 isolates from carriage and disease. Prevalence of antimicrobial resistance genes was overall lower in disease compared to carriage isolates, but increased significantly over time, mediated by rise in prevalence of a multidrug resistance element ICESag37 among disease isolates.

**Conclusion:**

There is a stable association between certain GBS genotypes and invasive disease, which suggests opportunities for developing more precise disease prevention strategies based on GBS targeted screening. In contrast, GBS mobile genetic elements appear less likely to be correlated with carriage or disease, and instead are associated with clonal expansion events across the GBS population.

## Introduction

*Streptococcus agalactiae* (Group B Streptococcus, GBS) is a common coloniser of the vaginal and gastrointestinal tracts of healthy adults. Carriage of GBS during pregnancy represents a risk factor for the development of invasive disease in the newborn and GBS is a leading cause of invasive infection in neonates worldwide (Gonçalves et al., 2022). Beta-lactams represent the first choice for intrapartum antibiotic prophylaxis (IAP) during labour and treatment of GBS disease. While most GBS isolates remain susceptible to beta-lactams (Kobayashi et al., 2021), prevalence of resistance to second-line antibiotics such as erythromycin and clindamycin has been increasing (Slotved and Hoffmann, 2020; Kekic et al., 2021; Sabroske et al., 2023).

Group B Streptococcus isolates are often grouped based on their capsular polysaccharide (CPS), with 10 different serotypes described to date: Ia, Ib, and II–IX (Berti et al., 2014). GBS CPS is a major virulence factor of GBS and a number of GBS multivalent vaccines targeting CPS are currently under development (Absalon et al., 2022). GBS isolates are also characterised using multi-locus sequence typing (MLST), which has revealed that five GBS clonal complexes (CCs) are associated with colonisation and disease in humans: CC1, CC10, CC17, CC19, and CC23 (Björnsdóttir et al., 2016; Khan et al., 2022). Some GBS lineages are associated with specific CPS serotypes, for instance CC17 isolates express predominantly serotype III (Teatero et al., 2016). Associations between GBS molecular markers and different host groups have been observed, with CC17-serotype III dominant among neonatal GBS invasive disease (Teatero et al., 2016; Bianchi-Jassir et al., 2020; Jamrozy et al., 2020), while CC1 often associated with disease in the adult population (Flores et al., 2015).

We have previously reported that CC17 prevalence has increased among GBS isolates from neonatal disease in the Netherlands, which was associated with expansion of particular CC17 clonal groups and with acquisition of a novel phage phiStag1 (Jamrozy et al., 2020). It has been unclear whether the increasing prevalence of these CC17 clones occurred only among the disease-associated GBS isolates, or was reflective of a more broad expansion across the GBS population. To address this, we have used whole genome sequencing (WGS) to analyse and contrast population structures of GBS isolates from maternal carriage and neonatal disease, collected in the Netherlands. Furthermore, to better understand the genetic variability between isolates from the two at-risk populations, we compared the distribution of key GBS molecular markers such as serotype, CC, antimicrobial resistance (AMR) genes and the intra-lineage population structure within the major CCs.

## Materials and methods

### GBS isolates

The collection consisted of 685 neonatal (<90 days old) invasive GBS isolates collected between 2006 and 2021, and 733 maternal carriage GBS isolates collected between 2017 and 2021 in the Netherlands. Isolates from neonatal disease were derived from a nationwide surveillance of bacterial meningitis and infant bacteraemia conducted by the Netherlands Reference Laboratory for Bacterial Meningitis (NRLMB). Disease isolates collected between 2006 and 2016 were described previously (Jamrozy et al., 2020). The infections were classified as early onset disease (EOD) at age 0–6 days, and as late onset disease (LOD) at age 7–89 days. Maternal carriage isolates were collected from pregnant women in hospitals in Amsterdam, The Hague, Utrecht, Hengelo, and Arnhem, for the Netherlands observational study on GBS disease, bacterial virulence and protective serology (NOGBS). Isolates were cultured from the vagina (*n* = 528) or urine (*n* = 205) according to local hospital protocols.

### Whole-genome sequencing and post processing

Genomic DNA was extracted using either the Wizard^®^ Genomic DNA Purification Kit or the Maxwell^®^ RSC Cultured Cells DNA Kit (AS1620) from Promega. Tagged DNA libraries were created using NEBNext^®^ Ultra™ II DNA Library Prep Kit for Illumina. Whole-genome sequencing was performed on the Illumina NovaSeq 6000 platform with 150 bp paired-end reads. Sequence reads were used to create assemblies using SPAdes v3.10.0 (Bankevich et al., 2012). Annotated assemblies were produced as described previously (Page et al., 2016).

### Whole-genome sequence data analysis

The sequence data was assessed using GBS QC pipeline v1.0.3^1^. Sequences that have passed QC were analysed using the GBS typer pipeline v1.0.10^2^ to determine sequence type (ST), serotype, and AMR gene carriage. Novel MLST alleles and ST profiles were deposited in the MLST database^3^. Isolates were assigned to a CC using the geoBURST algorithm in PHYLOVIZ v2.0 (Nascimento et al., 2017) and a single locus variant for group definition. To determine the presence of a phiStag1 (Jamrozy et al., 2020) and ICESag37 elements, sequence reads were mapped to reference sequences (phiStag1: GenBank accession PP091924; ICESag37: accession no. CP019978, 629058-702486) with SRST2 v0.2 using default parameters (Inouye et al., 2014).

Phylogenetic analyses were performed as detailed in Supplementary Methods. CC17 isolates from the Netherlands were supplemented with publicly available CC17 genomes to reconstruct a global, time-calibrated phylogeny as detailed in Supplementary Methods.

### Statistical analysis

Fisher’s exact test was used to determine significant association between host status and GBS genotypes, *P*-value < 0.001 was considered statistically significant.

## Results

### Serotype, ST, and CC distribution among GBS from carriage and disease

The dataset consisted of 733 maternal carriage and 685 neonatal disease isolates. The majority of neonatal isolates were from EOD (62%) with the remainder derived from LOD (38%; Supplementary Table 1).

**FIGURE 1:**
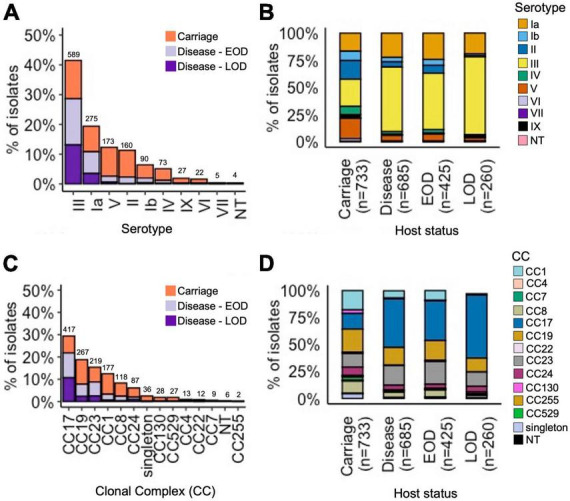
Serotype and CC distribution among the GBS isolates by host status. **(A)** Proportion of all isolates representing each serotype, stratified by host status. **(B)** Relative serotype distribution in each host status group. **(C)** Proportion of all isolates representing each CC, stratified by host status. **(D)** Relative CC distribution in each host status group. **(A,C)** Total number of isolates for each serotype and CC, respectively, is displayed above the bars.

Based on the *in silico* analysis, nine capsular serotype genotypes were identified (Ia, Ib, II–VII, and IX), while six isolates were non-typeable (Figure 1A). The most common serotypes among carriage isolates were III (25%), V (19%), II (17%), and Ia (17%), while disease isolates were predominantly serotype III (59%), followed by Ia (22%) (Figure 1B).

We identified 149 unique STs (Supplementary Table 1). The most common STs among carriage isolates were: ST1 (11%), ST17 (11%), ST23 (9%), ST19 (9%), ST28 (6%), and ST24 (5%) (Supplementary Figure 1). In contrast, disease isolates were dominated by ST17 (39%), followed by less common ST23 (15%) and ST19 (10%). The STs were grouped into 12 CCs. The main CCs among all GBS isolates were CC17 (29%), CC19 (19%), CC23 (15%), CC1 (13%), and CC8 (8%) (Figure 1C). In line with ST assignment, the majority of disease isolates belonged to CC17 (45%), followed by CC23 (18%) and CC19 (16%). The carriage isolates showed a more diverse CC distribution, spread across the five main CCs: CC19 (21%), CC1 (18%), CC17 (15%), CC23 (13%), and CC8 (11%) (Figure 1D).

**FIGURE 2:**
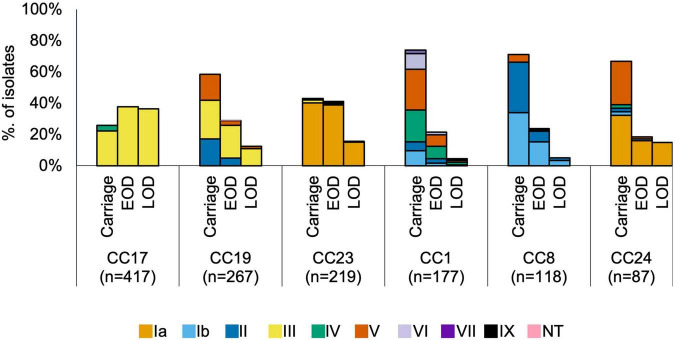
Serotype distribution by host status and CC. EOD, early onset disease; LOD, late onset disease.

We analysed associations between CCs and serotypes (Figure 2 and Supplementary Figure 2), which showed that CC17 and CC23 carried a single dominant serotype, III and Ia, respectively, while the other main CCs had a higher serotype diversity (Figure 2). Most serotypes were associated with multiple CCs, except for VI and VII which were only identified in CC1, while serotype IX was found only in CC130 isolates (Supplementary Figure 2).

We wished to compare the distribution of genotypes between isolates from carriage and disease. However, since our dataset was not fully temporally matched, we needed to account for the possibility of sampling bias due to the previously reported temporal changes in the prevalence of certain GBS lineages among isolates from neonatal invasive disease in the Netherlands (Jamrozy et al., 2020). To account for the likelihood of a continuing temporal trend in frequency of GBS genotypes, we have evaluated the differences between carriage and disease isolates by comparing a full dataset as well as a subset consisting only of isolates that were collected during overlapping collection years (2018–2021). As such, the latter included only the most recently collected disease isolates.

Across the full dataset we observed that serotype III was significantly more common in disease while serotypes Ib, II, IV, and V were more prevalent in carriage isolates (*P* < 0.001; Table 1). Among temporarily matched datasets, serotypes II, IV, and V remained more common in carriage although this was not statistically significant, while serotype III was still significantly associated with disease isolates.

**TABLE 1:** Prevalence of genotypes found to be differentially distributed between GBS from carriage and disease.

	Full			Time-matched subset		
	Carriage	Disease	*P*-value	Carriage	Disease	*P*-value
**Serotype**						
Ib	8% (62)	4% (28)	<0.001	8% (59)	9% (6)	1
II	17% (127)	5% (33)	<0.001	17% (120)	6% (4)	0.01
III	25% (183)	59% (406)	<0.001	25% (177)	57% (40)	<0.001
IV	8% (56)	2% (17)	<0.001	8% (53)	0	0.01
V	19% (137)	5% (36)	<0.001	19% (130)	6% (4)	0.004
**MLST**						
ST1	11% (77)	3% (23)	<0.001	10% (71)	3% (2)	0.05
ST17	11% (82)	39% (266)	<0.001	11% (80)	43% (30)	<0.001
ST28	6% (41)	1% (7)	<0.001	5% (38)	1% (1)	0.25
ST291	2% (15)	0	<0.001	2% (12)	0	0.61
ST569	2% (15)	0	<0.001	2% (15)	0	0.38
**CC**						
CC1	18% (131)	7% (46)	<0.001	18% (125)	3% (2)	<0.001
CC8	11% (84)	5% (34)	<0.001	11% (80)	6% (4)	0.16
CC17	15% (108)	45% (309)	<0.001	15% (103)	53% (37)	<0.001
CC19	21% (156)	16% (111)	0.02	21% (149)	6% (4)	<0.001
**CC-serotype**						
CC17-IV	2% (15)	0	<0.001	2% (12)	0	0.04
CC24-V	3% (24)	0.1% (1)	<0.001	3% (24)	1% (1)	0.63
**Clades**						
CC1-A	9% (68)	3% (22)	<0.001	9% (64)	3% (2)	0.07
CC8-C	5% (37)	2% (11)	<0.001	5% (34)	3% (2)	0.76
CC17-A0	0.4% (3)	7% (50)	<0.001	0.4% (3)	9% (6)	<0.001
CC17-A1	5% (37)	24% (167)	<0.001	5% (37)	21% (15)	<0.001
CC17-B	2% (13)	5% (36)	<0.001	2% (12)	9% (6)	0.004
CC19-A	1% (8)	7% (46)	<0.001	1% (8)	3% (2)	0.23
CC19-B	13% (94)	6% (42)	<0.001	13% (90)	1% (1)	0.002
CC19-D	6% (45)	1% (7)	<0.001	6% (42)	1% (1)	0.17
**MGE**						
phiStag1	21% (156)	32% (218)	<0.001	21% (148)	39% (27)	0.002
**AMR**						
MLS_B_	24% (179)	15% (102)	<0.001	25% (172)	26% (18)	0.88
ICESag37	4% (31)	5% (37)	0.27	4% (30)	17% (12)	<0.001

The total number of isolates from carriage/disease with corresponding genotype is shown in brackets. The prevalence of genotypes is shown for the full and time-matched (2018–2021) datasets.

Among all isolates, ST17 was significantly more common in disease, while ST1, ST28, ST291, and ST569 were significantly associated with carriage isolates (*P* < 0.001; Table 1). In time-matched datasets, these carriage-associated STs were still more prevalent among carriage isolates but this was not statistically significant. In contrast, ST17 was still significantly more common among disease isolates. In line with these associations, CC17 was significantly associated with disease while CC1 with the carriage isolates (*P* < 0.001), which was observed across the full and time-matched datasets. Additionally, CC8 isolates were more common in carriage although this was statistically significant only for the full dataset. We also observed that CC19 was significantly (*P* < 0.001) more common in carriage but only within the time-matched dataset, due to a substantial drop in its prevalence in the most recent disease isolates. Regarding CC-serotype associations, isolates from CC24-serotype V and CC17-serotype IV were found exclusively in carriage isolates except for a single CC24-serotype V identified in disease isolate (Table 1).

We also compared the distribution of genotypes between maternal carriage isolates collected from vagina and urine and observed no variation in prevalence of serotypes and CCs between the two isolation sources (Supplementary Figure 3).

### Phylogenetic structure and host status associations within GBS CC

**FIGURE 3:**
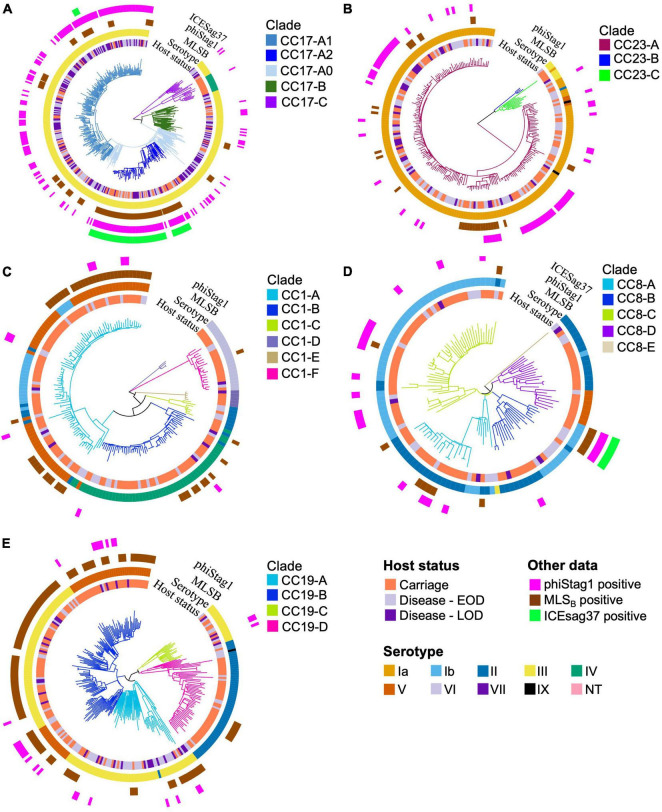
Phylogenetic trees of the five major CCs. The branches of each tree are coloured in accordance with CC-specific clusters ID. Each tip is annotated with (from the innermost circle): host status, serotype, carriage of MLS_B_ resistance genes, phiStag1 and ICESag37 (where applicable). Phylogenetic trees of **(A)** CC17, **(B)** CC23, **(C)** CC1, **(D)** CC8, and **(E)** CC19.

**FIGURE 4:**
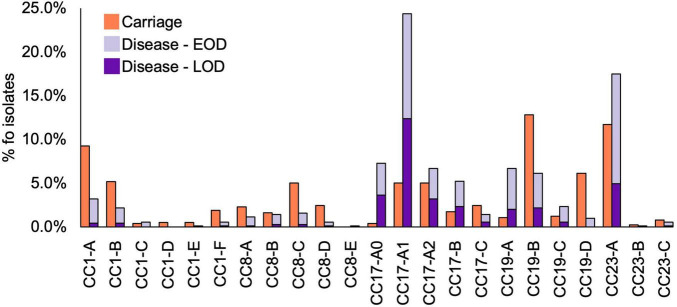
Prevalence of CC1, CC8, CC17, CC19, and CC23 clades among GBS isolates by host status. Disease isolates are stratified by disease onset (EOD, early onset disease; LOD, late onset disease).

Intra-lineage population structure was analysed by clustering each of the five major GBS CCs into phylogenetic clades (Figure 3). We have previously reported a clonal expansion of specific CC17 clades (CC17-A1 and CC17-A2) among GBS isolates from neonatal disease in the Netherlands (Jamrozy et al., 2020) and wished to compare their distribution among carriage and disease isolates, together with a broader comparison of GBS population between the two host groups. The phylogenetic trees of CC17 and CC23 revealed a single dominant clade (CC17-A and CC23-A, respectively), while the phylogenies of other CCs were more diverse, revealing between 4 and 6 distinct clades each. To identify the CC17 clades associated with the previously reported expansion, the dominant CC17 clade, CC17-A, was partitioned further into three sub-clades: CC17-A1, CC17-A2, and CC17-A0. For each clade identified, we calculated its prevalence across all carriage and disease isolates to identify dominant clusters within each host group and to compare their distribution (Figure 4).

The most common clades among the carriage isolates were CC19-B (13%), CC23-A (12%), and CC1-A (9%). In disease isolates, the most prevalent clades were CC17-A1 (24%) and CC23-A (18%). Additionally, clades CC1-A, CC8-C, CC19-B, and CC19-D were significantly more common among carriage while CC17-A0, CC17-A1, CC17-B, and CC19-A were associated with the disease isolates (*P* < 0.001; Table 1). Those associations remained significant in time-matched datasets only for CC17-A0 and CC17-A1.

To better understand the variable CC clade distribution between carriage and disease isolates, we also compared the prevalence of these clades within corresponding CC (Supplementary Figure 4). This has revealed that for CC1, CC8, and CC23 the distribution of clades was similar between carriage and disease isolates. For instance, CC1-A, CC8-C, and CC23-A represented dominant CC1, CC8, and CC23, respectively, clades in both carriage and disease. In contrast, for CC17 and CC19, we observed that variable clade distribution was associated with differences in CC17 and CC19 population structure between carriage and disease. As such, CC17-A1 was the dominant CC17 clade in disease isolates, while CC17 isolates from carriage showed an equal distribution of CC17-A1 and CC17-A2. The dominant CC19 clade in carriage isolates was CC19-B, while in disease the majority of isolates belonged to CC19-A.

Previous analysis of CC17 isolates from neonatal invasive disease in the Netherlands also revealed acquisition of a novel phage, phiStag1 (GenBank accession PP091924), which correlated with the clonal expansion of clade CC17-A1 (Jamrozy et al., 2020). In the current dataset, phiStag1 phage was found in 26% of all isolates, and it was significantly more common in disease (32%) in comparison to carriage (21%) isolates (Table 1). The phage was found predominantly in CC17 isolates where it was mostly associated with CC17-A1 and CC17-A2 (Supplementary Figure 5). Despite being more common in disease isolates overall, the phage was equally distributed among CC17 isolates from carriage and disease (Supplementary Figure 5). The phiStag1 phage was also detected in other dominant CCs: CC19 (10%), CC23 (28%), CC1 (5%), and CC8 (19%), where it was mostly equally distributed between carriage and disease (Supplementary Figure 5).

### GBS resistome

Tetracycline resistance genes (*tet*M, *tet*O, and *tet*L) were the most prevalent AMR determinants, observed in 86% of all GBS isolates. They were equally represented in disease and carriage isolates (Supplementary Table 2).

The second most common were genes conferring resistance to macrolides, lincosamides, and streptogramin B (MLS_B_) antibiotics (*erm*B, *erm*A, *erm*T, *mef*A*/msr*D, *lnu*B, *lsa*C, and *lsa*E), which were present in 20% of all GBS isolates (Supplementary Table 2). The most common MLS_B_ resistance determinants were *ermB* (12%), *mef*A/*msr*D (4%), and *erm*A (4%; Supplementary Table 2). Across the collection, the highest prevalence of MLS_B_ resistance genes was observed in isolates belonging to CC19 (32%), CC1 (30%), and CC17 (19%) (Supplementary Table 2), and the majority were from clades CC19-B, CC1-A and CC17-A2, respectively (Figures 3A, C, E). Across the full dataset, MLS_B_ resistance genes were more common in carriage (24%) in comparison to disease (15%) isolates. This was no longer observed in a time-matched dataset, which showed a comparable frequency of MLS_B_ resistance genes in carriage (25%) and disease isolates (26%).

Overall, 6% of all GBS isolates carried aminoglycoside resistance genes, with similar prevalence in isolates from carriage (7%) and disease (6%) in a full dataset (Supplementary Table 2). However, in a time-matched dataset they became more common in disease isolates (17%). Low frequency of chloramphenicol resistance genes (1%) was observed, mostly in carriage isolates (2%; Supplementary Table 2).

In CC17, a number of AMR determinants [*ant*(6-Ia), *aph*(3′-III), *aad*E, *erm*B, *tet*O] were carried by clonally related isolates (Supplementary Figure 6). Further analysis revealed that these resistance genes were located on a single, previously defined mobile genetic element (MGE), ICESag37 (Zhou et al., 2017). The majority of CC17 isolates carrying ICESag37 belonged to CC17-A2 (94%; Figure 3A). The ICESag37 element was also detected in CC8 isolates, exclusively in clade CC8-B (Figure 3D). The prevalence of ICESag37 was similar in carriage (4%) and disease (5%) isolates in a full dataset. However, its prevalence increased substantially in more recent disease isolates and in a time-matched dataset it was significantly more prevalent in disease (*P* < 0.001; Table 1).

### Global CC17 phylogeny and prevalence of ICESag37

**FIGURE 5:**
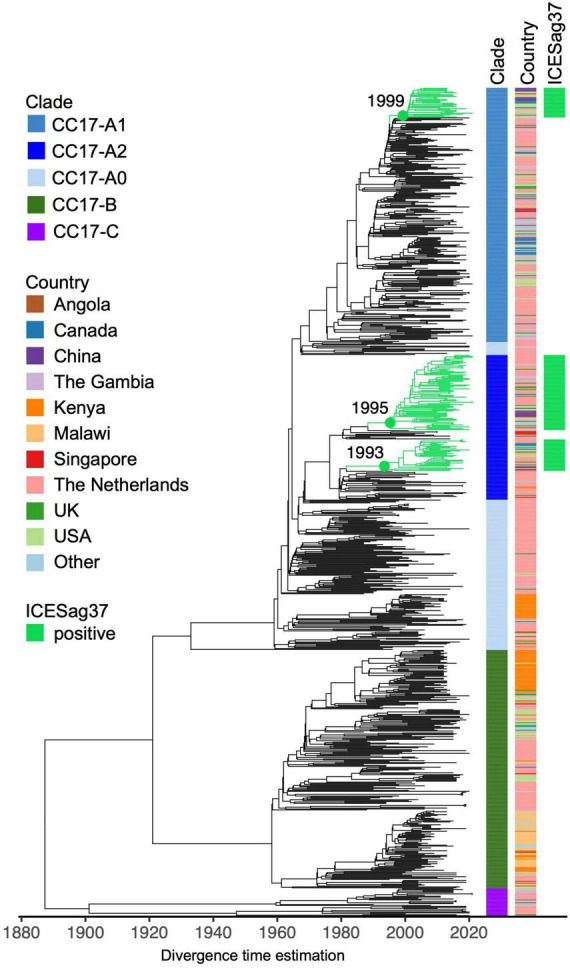
Core genome time-calibrated maximum likelihood phylogeny of global CC17 GBS isolates. The tree consists of external (*n* = 650) and Dutch (*n* = 229) CC17 GBS genomes. Each tip is annotated with CC17 clade ID and country of isolation (“Other”: countries represented by less than 10 isolates). Branches of clusters carrying ICESag37 are coloured in green.

To further investigate the apparent association between ICESag37 element and CC17-A2 isolates, we combined our CC17 sequence data (*n* = 229) with publicly available CC17 genomes (*n* = 650) (Supplementary Table 3) and reconstructed a time-calibrated, global CC17 phylogeny (Figure 5). The non-Dutch CC17 isolates represented 19 countries and most were derived from disease (83%; Supplementary Table 3).

The global CC17 isolates clustered into the three previously observed clades: CC17-A, CC17-B, and CC17-C (Figure 5). The majority of CC17-A isolates were represented by clade CC17-A1 (45%). The ICESag37 element was identified in 10% of non-Dutch CC17 genomes and only in isolates belonging to CC17-A, predominantly in CC17-A2 (63%) but also in CC17-A1 (12%) (Figure 5 and Supplementary Figure 7). The ICESag37-positive CC17 isolates were globally distributed and clustered into three distinct sub-clades, indicating multiple independent acquisition events followed by clonal expansion (Figure 5 and Supplementary Figure 7). It was estimated that all ICESag37-positive sub-clades emerged in the 1990s. Based on this dataset, the first ICESag37 positive CC17 isolates were collected in 2010 in Canada and China, with the first isolation in the Netherlands in 2011 (Supplementary Figure 8). Regardless of the country of origin, the majority of globally derived CC17-A2 isolates collected between 2010 and 2021 were ICESag37 positive (Supplementary Figure 8). ICESag37 sequence from all globally distributed CC17 isolates shared significant nucleotide identity (93%–100%, median 99.8%) (Supplementary Figure 9).

## Discussion

Intrapartum antibiotic prophylaxis currently represents the main strategy for the prevention of early onset GBS disease. This prevention strategy assumes an equal risk of neonatal invasive disease from any identified colonising GBS isolate. However, our and previous research clearly showed that some GBS genotypes carry a higher risk of neonatal disease. More studies are needed to investigate the pathophysiological mechanisms that drive these differences in invasive potential and evaluate the added value of GBS genotype determination to more precisely target GBS prevention. Our work has shown that, in line with previous reports, CC17-serotype III strains were significantly more common in disease (Kekic et al., 2021). while serotypes II, IV, V, and CC1 were associated with maternal carriage. We have also identified variable prevalence of some lineage-serotype combinations between the two host groups. This included isolates representing CC24-serotype V and CC17-serotype IV, which were associated with carriage. This suggests that the association between CC17 and neonatal disease is serotype III dependent. Although other serotypes have emerged within this GBS lineage, they appear less likely to cause neonatal infection as none of the CC17-serotype IV were observed among disease isolates in our collection. In contrast, serotype III remained associated with neonatal disease even after exclusion of all CC17 isolates (*P* < 0.001).

Our previous work has shown expansion of specific CC17 sub-clades, CC17-A1 and CC17-A2, among isolates from neonatal invasive disease in the Netherlands, which correlated with a rise in disease incidence in the country. A matched collection of isolates from maternal carriage from the Netherlands was not previously available, which hindered further investigation of the epidemiology of these clones in a wider GBS population. In this work, we addressed this data gap and compared the prevalence of different clades from major CCs, including CC17, between carriage and disease isolates. Overall, CC17-A1 clade was the most prevalent sub-lineage among all disease isolates, suggesting an increased capacity to cause disease. However, although it was considerably less common among all carriage isolates, the CC17 population from carriage was dominated by CC17-A1 and CC17-A2 isolates. This suggests that the previously reported rise in the frequency of these clusters in GBS from neonatal disease likely reflected their expansion in the carriage GBS CC17 population, which resulted in a spillover to invasive GBS population.

We also reported previously and in this work that the expanding CC17 sub-clades, CC17-A1 and CC17-A2, are associated with certain MGEs that might contribute to their prevalence. One is a novel phage, previously termed phiStag1, which emerged suddenly in the CC17 population around the mid-1990s (Jamrozy et al., 2020). A recent study has shown that the phage belongs to a novel group of phages designated streptococcal mobilisable prophages (SMphages) (Huang et al., 2023). The phage carries a putative virulence gene, which was termed Alp-P1 and was shown to promote the adhesion and invasion of bovine and human cells. These findings further indicate that phiStag1 might provide some selective advantage to its host and thus promote clonal expansion of CC17-A1 and CC17-A2. In our dataset, we found phiStag1 to be overall more common among disease isolates. However, among CC17 isolates, the phage was equally distributed among carriage and disease. Further work is needed to better understand phiStag1’s role in GBS disease. While it was found more common in isolates from disease, this was likely driven by its association with CC17 and the dominance of this lineage within disease. It remains unclear if presence of this phage contributes to maternal colonisation, transmission to the infant or neonatal invasive disease.

We have also observed a high prevalence of the ICESag37 element among CC17 isolates. This MGE confers resistance to erythromycin, tetracycline and aminoglycosides (Zhou et al., 2017). It was first identified in the Sag37 strain, which represents ST12. In our dataset, ICESag37 was most common in CC17 (15%), followed by CC8 (4%), which includes ST12. Carriage of a MDR ICESag element, corresponding to ICESag37, has been reported previously in CC17 (Campisi et al., 2016). Our analysis of a global CC17 phylogeny has confirmed that ICESag37-positive CC17 isolates are widely distributed and have been found in Asia, Europe, and North America. We also observed that carriage of this MGE within CC17 is associated mostly with sub-clade CC17-A2. Within the Dutch GBS collection, CC17-A2 accounted for 87% of all isolates carrying ICESag37. As such, ICESag37-positive CC17-A2 isolates resistant to both macrolides and aminoglycosides might pose a clinical threat due to reduced options for first- and second-line antimicrobial treatment of GBS infections.

Limitations of our study include a temporal sampling bias, with disease and carriage isolates collected over different time periods, with only a 4-year overlap between the two collections (2018–2021). To account for this, we conducted a parallel analysis of full and time-matched datasets. While some genotypes showed statistically significant associations across both datasets, for many the differences between carriage and disease isolates were no longer statistically significant in time-matched dataset, which is likely partly due to much lower disease sample size in the latter. However, the analysis also showed that the prevalence of AMR genes was higher in most recently collected disease isolates, which was associated with increase in frequency of isolates carrying the ICESag37 element. Finally, the maternal carriage isolates were recovered from vagina and urine, with the latter potentially associated with asymptomatic bacteriuria. However, we observed no variation in genotype distribution between isolates from these sources suggesting that GBS isolates from urine are acquired from the rectovaginal site and represent the same GBS population.

Here we report that the previously observed clonal expansion of CC17-A1 and CC17-A2 clades as well as the emergence of phiStag1 phage among GBS isolates from neonatal invasive disease in the Netherlands likely reflect changes in the maternal carriage population. Overall, our findings reinforce the importance of comparing GBS isolates from healthy individuals and patients to identify pathogen genotypes that might be associated with increased capacity to cause disease. Altogether this will provide pathogenicity markers that can be targeted in disease prevention strategies as well as molecular markers for surveillance of high-risk clones that demonstrate enhanced dissemination across GBS population irrespective of the host status.

## Data availability statement

All GBS isolates analyzed in this study are publicly available. The ENA accession numbers for each GBS isolate are provided in Supplementary Table 1.

## Ethics statement

Ethical approval was not required for the studies involving humans because it involved the analysis of anonymized whole genome sequence data from Group B Streptococcus (GBS) isolates collected in the Netherlands. The data used in this research were obtained from a collection database maintained by the Netherlands Reference Laboratory for Bacterial Meningitis, which serves as the national reference laboratory for community-acquired invasive bacterial infections, including neonatal GBS infections. As the data were anonymized and did not contain any identifiable patient information, the study did not involve direct interaction with human subjects. Furthermore, the research adhered to all relevant ethical guidelines and regulations governing the use of anonymized genomic data for scientific research purposes. Therefore, ethical approval was not deemed necessary for this type of retrospective, anonymized data analysis study. The studies were conducted in accordance with the local legislation and institutional requirements. Written informed consent for participation was not required from the participants or the participants’ legal guardians/next of kin in accordance with the national legislation and institutional requirements because in accordance with guidance from the Netherlands Reference Laboratory for Bacterial Meningitis, patient data utilized in this study were obtained from an anonymized collection database. As a result, individual patients are not identifiable or traceable, aligning with Dutch Law requirements.

## Author contributions

UK: Data curation, Formal analysis, Investigation, Methodology, Visualization, Writing – original draft. VD: Software, Writing – review & editing. CC: Methodology, Writing – review & editing. NS: Conceptualization, Writing – review & editing. DB: Conceptualization, Writing – review & editing. WM: Methodology, Writing – review & editing. SB: Conceptualization, Writing – review & editing. MB: Conceptualization, Writing – review & editing. DJ: Conceptualization, Methodology, Project administration, Supervision, Validation, Writing – review & editing.

## Members of the NOGBS study group

Eveline van Asbeck (Department of Obstetrics and Gynaecology, Tergooi Hospital, Hilversum, Netherlands), Rolanda Baars (Department of Paediatrics, St. Jansdal Hospital, Harderwijk, Netherlands), Desireé V. Ballegoy (Department of Medical Microbiology and Immunology, St. Antonius Hospital, Nieuwegein, Netherlands), Ron van Beek (Department of Paediatrics, Amphia Hospital, Breda, Netherlands), Vincent Bekker (Department of Paediatrics, Leiden University Medical Centre, Leiden, Netherlands), Maartje van den Berg (Department of Paediatrics, Haaglanden Medical Centre, The Hague, Netherlands), Geert Jan Blok (Department of Neonatology, Northwest Clinics, Alkmaar, Netherlands), Mijke Breukels (Department of Paediatrics, Elkerliek Hospital, Helmond, Netherlands), Alwin F. J. Brouwer (Department of Paediatrics, Hospital of Nij Smellinghe, Drachten, Netherlands), Matthijs Brouwer (Department of Neurology, Amsterdam UMC, Amsterdam, Netherlands), Renske Cornelisse-van Vugt (Department of Paediatrics, Canisius Wilhelmina Hospital, Nijmegen, Netherlands), Dick van Dam (Laboratory for Medical Microbiology and Public Health, Hengelo, Netherlands), Karin van Dijk (Department of Medical Microbiology and Infection Control, Amsterdam University Medical Centre, Amsterdam Infection and Immunity Institute, Amsterdam, Netherlands), Luçan C. Delemarre (Department of Paediatrics, Amstelland Hospital, Amstelveen, Netherlands), Anouk Dings (Department of Paediatrics, Gelre Hospitals, Apeldoorn, Netherlands), Rienus A. Doedens (Department of Paediatrics, Martini Hospital, Groningen, Netherlands), Stefan M. van Dorth (Department of Paediatrics, Tjongerschans Hospital, Heerenveen, Netherlands), Gertjan Driessen (Department of Paediatrics, Haga Hospital, The Hague, Netherlands), Erika van Elzakker (Department of Medical Microbiology and Infection Prevention, Amsterdam University Medical Centre, University of Amsterdam, Amsterdam, Netherlands), Arie van der Ende (Department of Medical Microbiology and Infection Prevention, Amsterdam University Medical Centre, University of Amsterdam, Amsterdam, Netherlands), Katja de Graaff (Department of Obstetrics and Gynaecology, Reinier de Graaf Hospital, Delft, Netherlands), Hester M. Havers (Department of Paediatrics, Alrijne Hospital, Leiderdorp, Netherlands), Jojanneke Heidema (Department of Paediatrics, St. Antonius Hospital, Utrecht, Netherlands), Marieke A. C. Hemels (Department of Neonatology, Isala Clinics, Zwolle, Netherlands), Maartje E. N. van den Heuvel (Department of Paediatrics, Onze Lieve Vrouwe Gasthuis, West Location, Amsterdam, Netherlands), Marion E. van Hoorn (Department of Obstetrics and Gynaecology, HagaZiekenhuis, Den Haag, Netherlands), Marlies van Houten (Department of Paediatrics, Spaarne Gasthuis, Haarlem, Netherlands), Flip van der Hulst (Department of Paediatrics, Zaans Medical Centre, Zaandam, Netherlands), Monique A. M. Jacobs (Department of Paediatrics, Slingeland Hospital, Doetinchem, Netherlands), Arieke Janse (Department of Paediatrics, Gelderse Vallei Hospital, Ede, Netherlands), Miranda de Jong (Department of Paediatrics, Albert Schweitzer Hospital, Dordrecht, Netherlands), Anton H. van Kaam (Department of Neonatology, Amsterdam University Medical Centre, University of Amsterdam, Amsterdam, Netherlands), Ageeth Kaspers (Department of Paediatrics, Medisch Spectrum Twente, Twente, Netherlands), Merel N. van Kassel (Department of Neurology, Amsterdam University Medical Centre, University of Amsterdam, Amsterdam, Netherlands), Anne A. M. W. van Kempen (Department of Paediatrics, Onze Lieve Vrouwe Gasthuis, East Location, Amsterdam, Netherlands), Kristine Klúčovská (Department of Paediatrics, Treant Hospital Group, Hoogeveen, Netherlands), Karen Korbeek (Department of Paediatrics, St. Jansdal Hospital, Harderwijk, Netherlands), René F. Kornelisse (Department of Paediatrics, Erasmus Medical Centre, Rotterdam, Netherlands), Taco W. Kuijpers (Department of Paediatric Immunology, Rheumatology and Infectious Diseases, Amsterdam University Medical Centre, University of Amsterdam, Amsterdam, Netherlands), Elisabeth van Leeuwen (Department of Obstetrics and Gynaecology, Amsterdam University Medical Centre, University of Amsterdam, Amsterdam, Netherlands), Ineke Linde (Department of Infectious Diseases, GGD Amsterdam, Amsterdam, Netherlands), Jeannette von Lindern (Department of Paediatrics, Groene Hart Hospital, Gouda, Netherlands), Karen van Mechelen (Department of Neonatology, Maastricht University Medical Centre, Maastricht), Netherlands), Clemens B. Meijssen (Department of Paediatrics, Meander Medical Centre, Amersfoort, Netherlands), Jeroen Noordzij (Department of Paediatrics, Reinier de Graaf Hospital, Delft, Netherlands), Annemarie Oudshoorn (Department of Paediatrics, Gelre Hospitals, Apeldoorn, Netherlands), Frans B. Plötz (Tergooi Hospital, Hilversum, Netherlands), Maarten Rijpert (Department of Paediatrics, Zaans Medical Centre, Zaandam, Netherlands), Maaike van Rossem (Department of Paediatrics, Rijnstate Hospital, Arnhem, Netherlands), Machteld van Scherpenzeel (Department of Paediatrics, Medical Centre Leeuwarden, Leeuwarden, Netherlands), Maarten Schijffelen (Laboratory for Medical Microbiology and Public Health, Hengelo, Netherlands), George Shabo (Department of Paediatrics, Hospital Group Twente, Twente, Netherlands), Jacqueline van der Sluijs (Department of Neonatology, Maxima Medical Centre, Veldhoven, Netherlands), Linde Snoek (Department of Neurology, Amsterdam UMC, Amsterdam, Netherlands), Jacqueline U. M. Termote (Department of Neonatology, University Medical Centre Utrecht, Utrecht, Netherlands), Gerdien A. Tramper-Stranders (Department of Paediatrics, Franciscus Gasthuis, Rotterdam, Netherlands), Gavin W. ten Tusscher (Department of Paediatrics, Dijklander Hospital, Hoorn, Netherlands), Saraa J. Vainio (Department of Medical Microbiology and Immunology, St. Antonius Hospital, Nieuwegein, Netherlands), Joost van de Ven (Department of Obstetrics and Gynaecology, Elkerliek Hospital, Helmond, Netherlands), Mirjam van Veen (Department of Paediatrics, Haga Hospital, The Hague, Netherlands), Marlies Vermaas (Department of Paediatrics, Admiraal de Ruyter Hospital, Goes, Netherlands), Marjoke Verweij (Department of Paediatrics, Viecuri Medical Centre, Venlo, Netherlands), Douwe H. Visser (Department of Neonatology, Amsterdam University Medical Centre, University of Amsterdam, Amsterdam, Netherlands), Karlijn C. Vollebregt (Department of Obstetrics and Gynaecology, Spaarne Gasthuis, Haarlem, Netherlands), Wouter J. de Waal (Department of Paediatrics, Diakonesse Hospital, Utrecht, Netherlands), Anne-Marie van Wermeskerken (Department of Paediatrics, Flevohospital, Almere, Netherlands), Janneke Wilms (Department of Paediatrics, BovenIJ Hospital, Amsterdam, Netherlands), Tom F. W. Wolfs (Department of Paediatrics, University Medical Centre Utrecht, Utrecht, Netherlands), Maurice G. A. J. Wouters (Department of Obstetrics and Gynaecology, Amsterdam University Medical Centre, University of Amsterdam, Amsterdam, Netherlands).

## Footnotes

1
https://github.com/sanger-bentley-group/GBS_QC_nf

2
https://github.com/sanger-bentley-group/GBS-Typer-sanger-nf

3
https://pubmlst.org/sagalactiae/
