# Supplementary material for: Genetic markers associated with host status and clonal expansion of Group B Streptococcus in the Netherlands
Source: Front Microbiol. 2024 Jul 10;15:1410651. doi: 10.3389/fmicb.2024.1410651 (PMC11266191; doi:10.3389/fmicb.2024.1410651)
Supplement: Supplementary file 1 [file Data_Sheet_1.DOCX]

# Supplementary methods

### **Phylogenetic reconstructions and clustering**

To construct species wide phylogeny of all GBS isolates, sequence reads were mapped against the NGBS128 reference genome (accession no. NZ_CP012480.1) using SMALT v0.7.4 (<https://www.sanger.ac.uk/science/tools/smalt-0>). Regions representing putative mobile genetic elements (MGEs) were masked using remove_blocks_from_aln (<https://github.com/sanger-pathogens/remove_blocks_from_aln>) and SNPs were identified with SNP-sites (1). Alignment positions with >5% uncalled variants were filtered out. This core genome alignment was then used to reconstruct an approximately maximum-likelihood tree using FastTree v2.1.10 (2).

To construct species wide phylogeny of all GBS isolates, sequence reads were mapped against the NGBS128 reference genome (accession no. NZ_CP012480.1) using SMALT v0.7.4 (<https://www.sanger.ac.uk/science/tools/smalt-0>). Variants were called using SAMtools v1.6 (3) and bcftools v1.6. Regions representing putative mobile genetic elements (MGEs) were masked using remove_blocks_from_aln (<https://github.com/sanger-pathogens/remove_blocks_from_aln>) and SNPs were identified with SNP-sites (1). Alignment positions with >5% uncalled variants were filtered out. This core genome alignment was then used to reconstruct an approximately maximum-likelihood tree using FastTree v2.1.10 (2).

To reconstruct CC-specific phylogenies, sequence reads were mapped against CC-specific *S. agalactiae* reference genomes: SS1 (CC1; accession no. NZ_CP010867.1), Sag37 (CC10; accession no. NZ_CP019978.1), NGBS128 (CC17; accession no. NZ_CP012480.1), H002 (CC19; accession no. NZ_CP011329.1), NEM316 (CC23; accession no. AL732656.1), and whole genome alignments were generated as described above. Each CC-specific alignment was screened for presence of putative recombination regions, which were identified and masked using gubbins v3.2.1 (4). The core genome alignment files were then used to generate the maximum likelihood (ML) phylogenetic tree of each CC using RAXML, as implemented in gubbins v3.2.1 (4). The phylogenies were partitioned using a method by Prosperi *et al*. (5) based on pairwise SNP distances and node reliability of ≥90% (bootstrap support). The phylogenetic trees were annotated and visualised using iTOL v4 (6).

To access publicly available CC17 genomes, 17636 paired end whole genome sequence data representing *S. agalactiae* (taxon ID 1311) was downloaded from ENA on November 3rd, 2022 and were run through GBS QC pipeline v1.0.3 (<https://github.com/sanger-bentley-group/GBS_QC_nf>) and GBS typing pipeline v1.0.10 (<https://github.com/sanger-bentley-group/GBS-Typer-sanger-nf>). Of the 17636 whole genome sequences, 16303 (92%) passed QC among which 14033 (86%) were derived from humans. CC17 comprised 2364 (17%) GBS isolates. Information on country and year of sample collection was available for 1625 isolates, which were selected for the further analysis. The presence of the ICESag37 element in the external CC17 dataset was screened using SRST2 v0.2 (7) and presence/absence was called based on a 90% sequence coverage cut-off.

To reconstruct the global, time-calibrated phylogeny of CC17, the external (n = 1625) and Dutch (n = 417) CC17 GBS isolates were randomly subsampled by selecting up to 30 isolates from each year and including all ICESag37 positive isolates. The Dutch CC17 (n=229) dataset and sub sampled external CC17 (n=650) isolates were mapped to NGBS128 reference genome (accession no. NZ_CP012480.1) to generate core genome alignment and maximum likelihood tree using the same methods described above for the CC-specific phylogenies. To reconstruct time-calibrated phylogeny, each sequence in the core genome alignment was annotated with the date of isolation based on year. Bayesian inference of phylogeny was performed with Bactdating (8) (<https://github.com/xavierdidelot/BactDating>) under a relaxed gamma clock model. The MCMC chains were run for 1e6 iterations.

1. Page AJ, Taylor B, Delaney AJ, Soares J, Seemann T, Keane JA, et al. SNP-sites: rapid efficient extraction of SNPs from multi-FASTA alignments. Microb Genom. 2016;2(4):e000056.

2. Price MN, Dehal PS, Arkin AP. FastTree 2--approximately maximum-likelihood trees for large alignments. PLoS One. 2010;5(3):e9490.

3. Li H, Handsaker B, Wysoker A, Fennell T, Ruan J, Homer N, et al. The Sequence Alignment/Map format and SAMtools. Bioinformatics. 2009;25(16):2078-9.

4. Croucher NJ, Page AJ, Connor TR, Delaney AJ, Keane JA, Bentley SD, et al. Rapid phylogenetic analysis of large samples of recombinant bacterial whole genome sequences using Gubbins. Nucleic Acids Res. 2015;43(3):e15.

5. Prosperi MC, Ciccozzi M, Fanti I, Saladini F, Pecorari M, Borghi V, et al. A novel methodology for large-scale phylogeny partition. Nat Commun. 2011;2:321.

6. Letunic I, Bork P. Interactive Tree Of Life (iTOL) v4: recent updates and new developments. Nucleic Acids Res. 2019;47(W1):W256-w9.

7. Inouye M, Dashnow H, Raven LA, Schultz MB, Pope BJ, Tomita T, et al. SRST2: Rapid genomic surveillance for public health and hospital microbiology labs. Genome Med. 2014;6(11):90.

8. Didelot X, Croucher NJ, Bentley SD, Harris SR, Wilson DJ. Bayesian inference of ancestral dates on bacterial phylogenetic trees. Nucleic Acids Res. 2018;46(22):e134.
