# Supplementary material for: Genetic markers associated with host status and clonal expansion of Group B Streptococcus in the Netherlands
Source: Front Microbiol. 2024 Jul 10;15:1410651. doi: 10.3389/fmicb.2024.1410651 (PMC11266191; doi:10.3389/fmicb.2024.1410651)
Supplement: Supplementary file 2 [file Data_Sheet_2.docx]

**Supplementary figures**


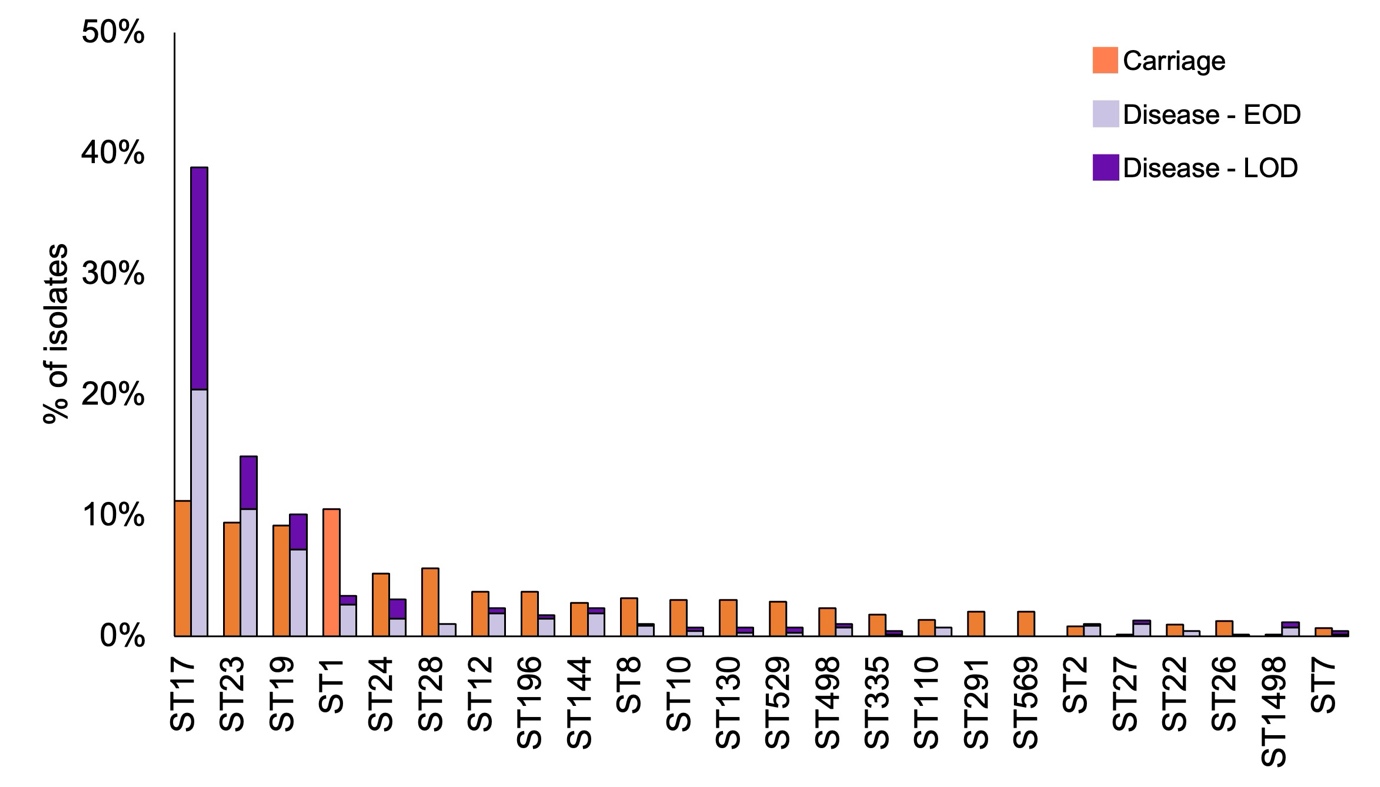


**Figure S1. ST distribution among carriage and disease isolates.** Disease isolates are stratified by disease onset (EOD: early onset disease, LOD: late onset disease). Only ST observed in more than 1% of total isolates are displayed.


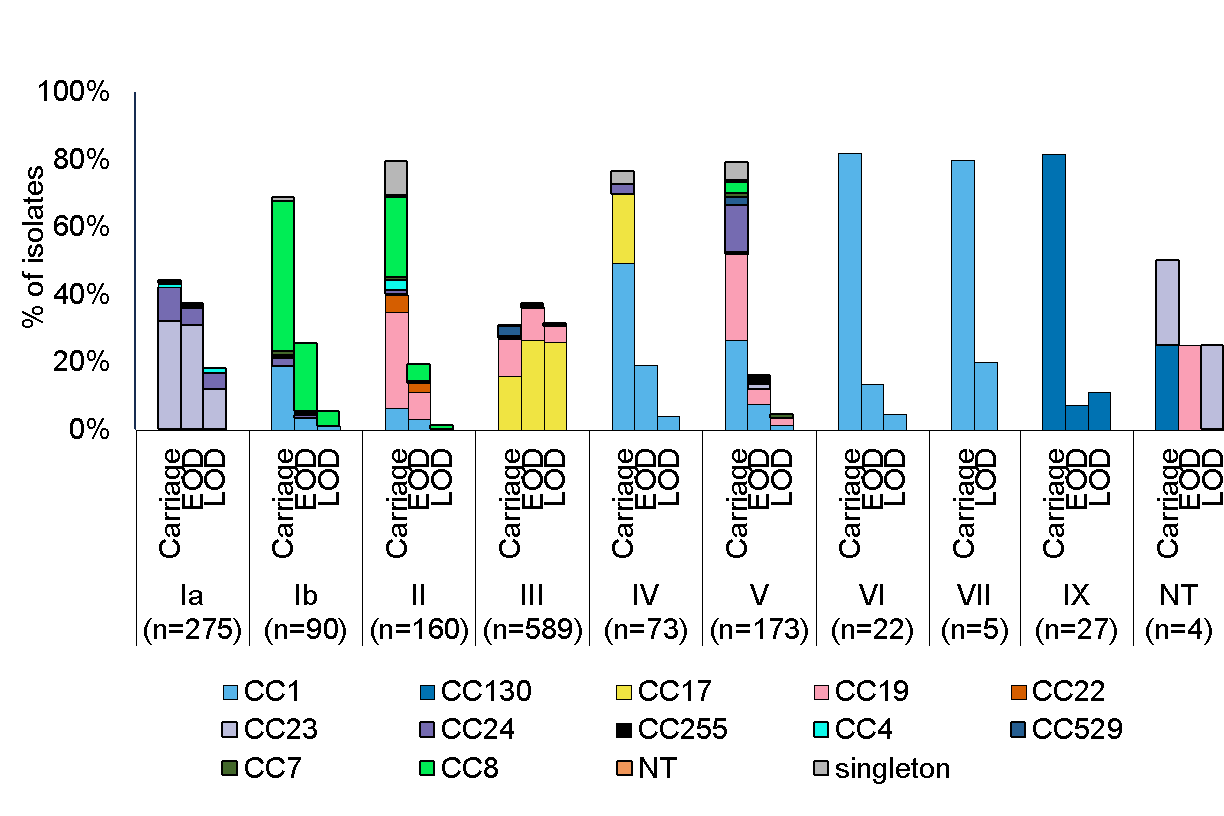


**Figure S2. CC distribution by host status and serotype.** EOD: early onset disease, LOD: late onset disease.


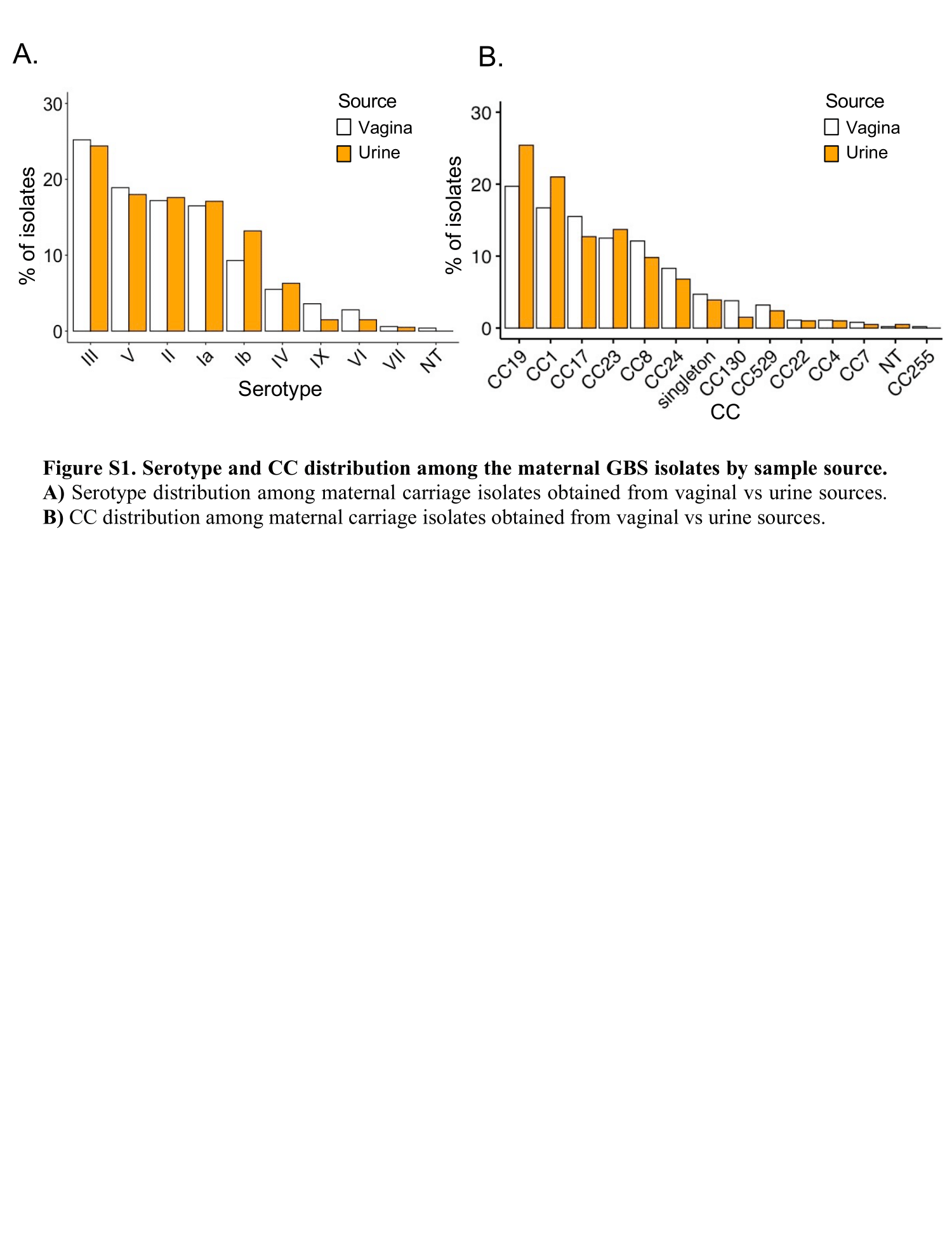


**Figure S3. Serotype and CC distribution among the maternal GBS isolates by sample source. A)** Serotype distribution and **B)** CC distribution among maternal carriage isolates obtained from vaginal (n = 528) vs urine (n = 205) sources.


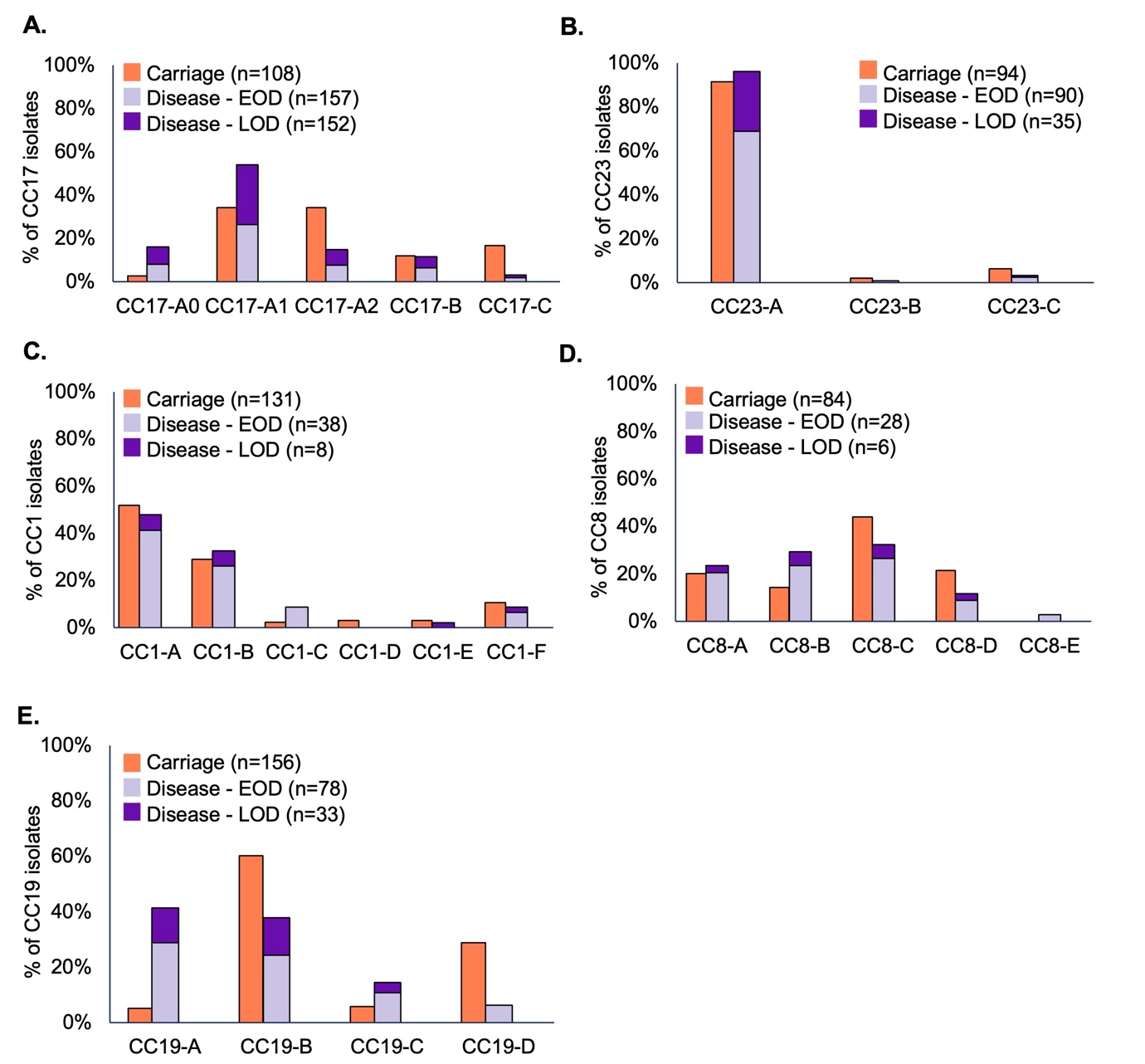


**Figure S4. Prevalence of CC1, CC8, CC17, CC19 and CC23 clades among GBS isolates within corresponding CC for each host status.** Disease isolates are stratified by disease onset (EOD: early onset disease, LOD: late onset disease). **A)** Prevalence of CC17 clades among CC17 isolates. **B)** Prevalence of CC23 clades among CC23 isolates. **C)** Prevalence of CC1 clades among CC1 isolates. **D)** Prevalence of CC8 clades among CC8 isolates. **E)** Prevalence of CC19 clades among CC19 isolates.


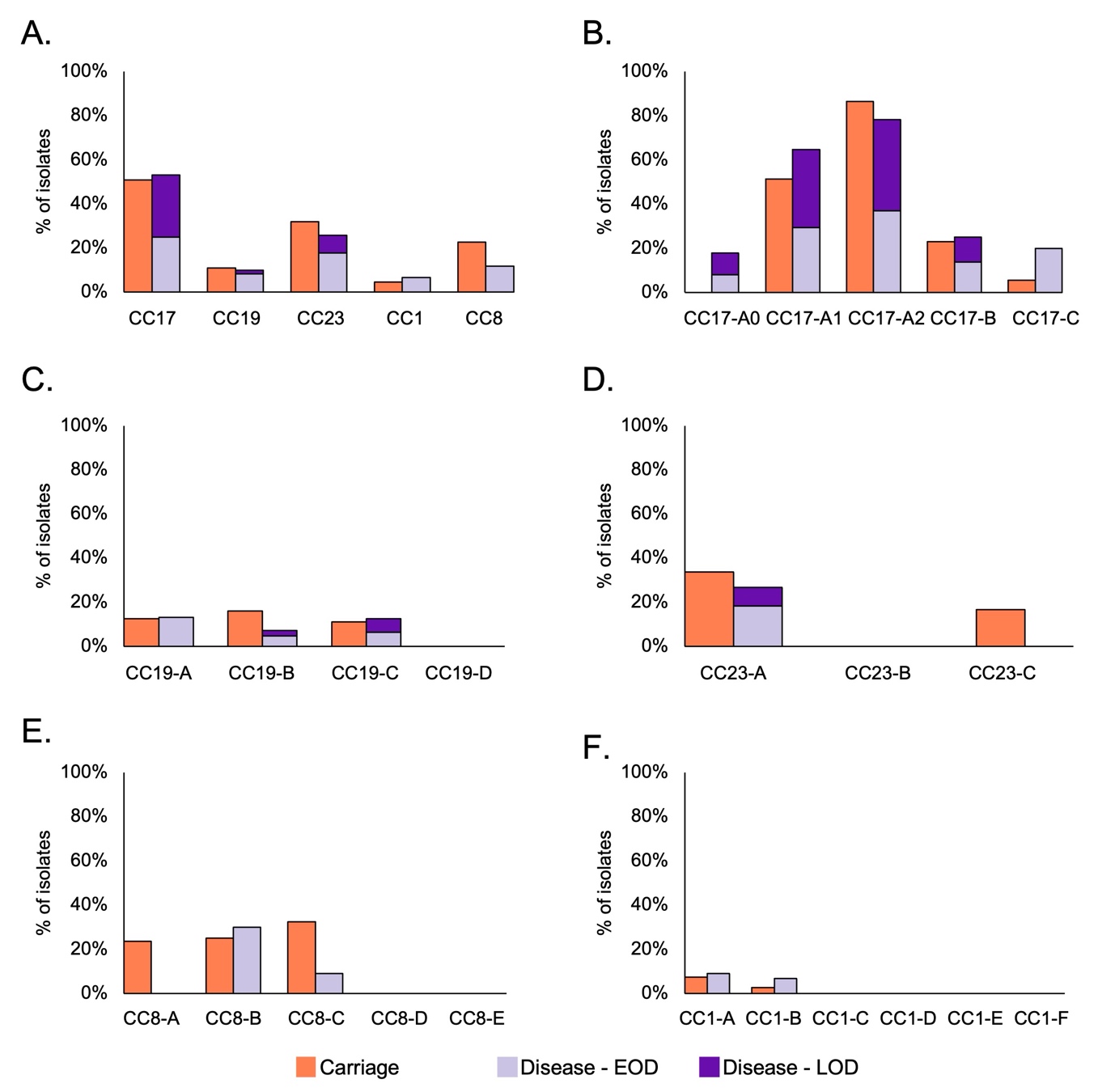


**Figure S5. Prevalence of the phiStag1 phage among carriage and disease isolates across the five main GBS CCs.** Disease isolates are stratified by disease onset (EOD: early onset disease, LOD: late onset disease). **A)** Prevalence of phiStag1 among five main GBS CCs. **B-F)** Prevalence of phiStag1 among different clades from **B)** CC17, **C)** CC19, **D)** CC23, **E)** CC8, and **F)** CC1.

**
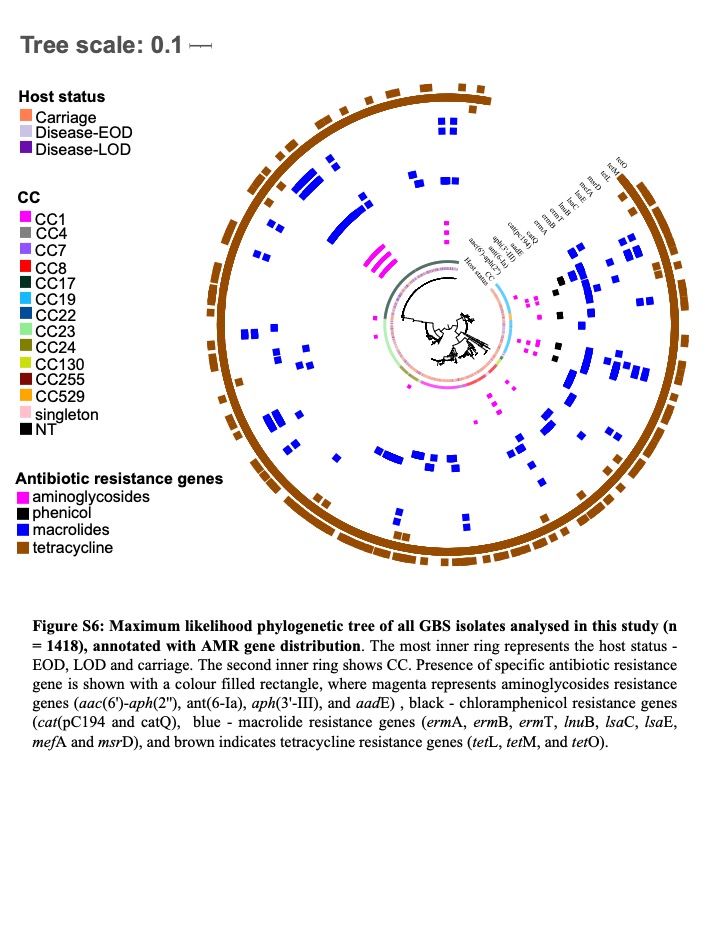
**

**Figure S6. Core genome maximum likelihood phylogenetic tree of all GBS isolates from the Netherlands analysed in this study**. Each tip is annotated with (from the innermost circle): host status, CC, presence of screened antibiotic resistance genes (*aac*(6')-*aph*(2''), ant(6-Ia), *aph*(3'-III), *aad*E​​, *cat*(pC194) catQ,  *erm*A, *erm*B, *erm*T, *lnu*B, *lsa*C, *lsa*E, *mef*A and *msr*D, *tet*L, *tet*M, and *tet*O.

**
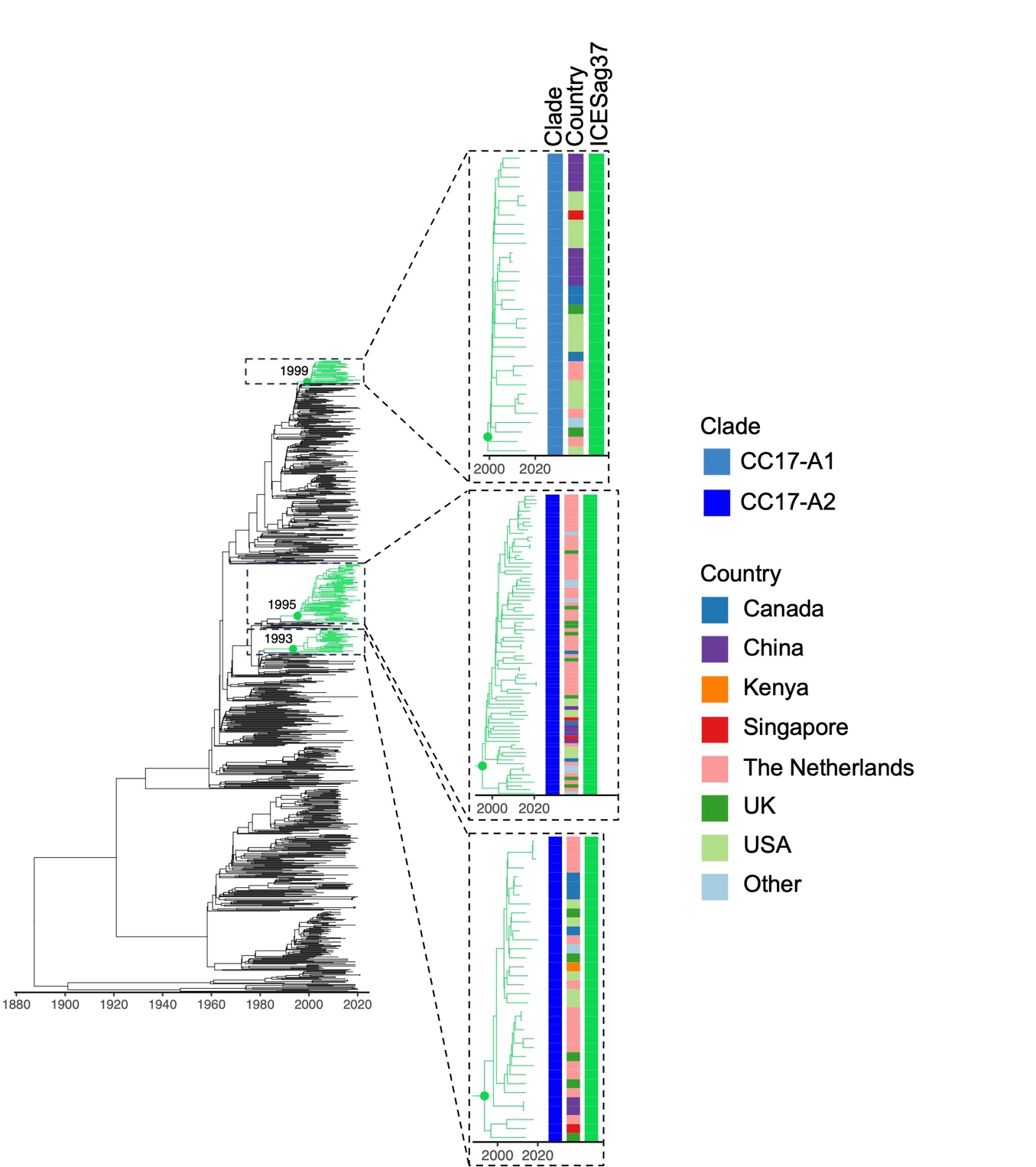
**

**Figure S7. Expanded view of global sub-clades CC17-A1 and CC17-A2 carrying ICESag37.** Tips of the tree are annotated with CC17 sub-clade ID and country of origin.

**
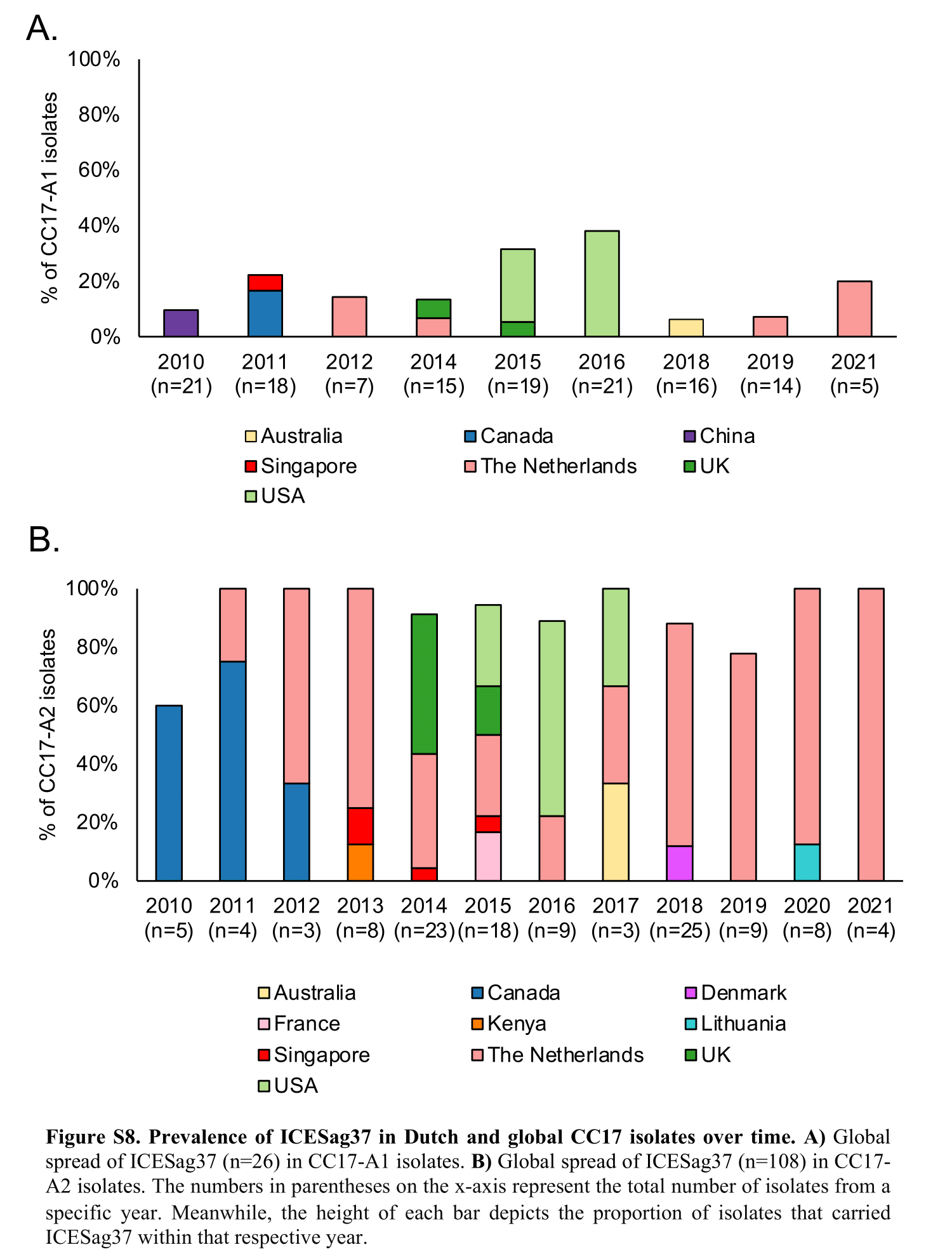
**

**Figure S8. Prevalence of ICESag37 in global CC17 subclade isolates over time.** Proportion of ICESag37 positive isolates for each year, stratified by country of origin in **A)** CC17-A1 **B)** CC17-A2 isolates. The total number of isolates available from each year is shown in brackets on the x axis.

**
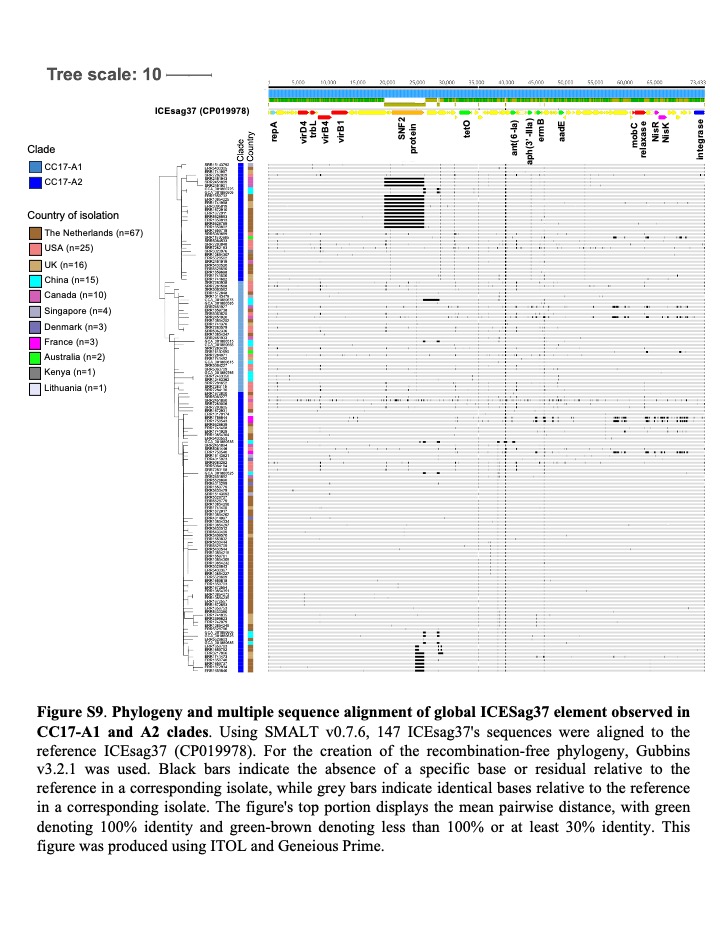
**

**Figure S9**. **Phylogeny and alignment of ICESag37 element sequences observed in global CC17-A1 and CC17-A2 isolates**. Each tip is annotated with genome sequence accession ID, CC17 sub-clade ID and isolate country of origin. Grey bars within the alignment plot indicate identical bases in a corresponding isolate while black bars indicate the absence of a specific base or residual relative to the reference. The top horizontal panel represents the ICESag37 reference sequence**.** The middle section represents the mean pairwise distance, with green denoting 100% identity and green-brown denoting less than 100% but over 30% identity.
