# Supplementary material for: Genetic markers associated with host status and clonal expansion of Group B Streptococcus in the Netherlands
Source: Front Microbiol. 2024 Jul 10;15:1410651. doi: 10.3389/fmicb.2024.1410651 (PMC11266191; doi:10.3389/fmicb.2024.1410651)
Supplement: Supplementary file 4 [file Table_2.DOCX]

**Table S2. Prevalence of AMR genes in the analysed GBS isolates by CC and host status.** The total number of isolates with corresponding AMR gene is shown in brackets. The prevalence of AMR genes by host status is shown for the full and time-matched (2018-2021) datasets.

|  |  |  |  |  |  |  | **Full** | | **Time-matched subset** | |
| --- | --- | --- | --- | --- | --- | --- | --- | --- | --- | --- |
|  | **Total** | **CC1** | **CC8** | **CC17** | **CC19** | **CC23** | **Carriage** | **Disease** | **Carriage** | **Disease** |
| TET^a^ | 86% (1221) | 76% (134) | 82% (97) | 96% (401) | 85% (226) | 89% (196) | 84% (617) | 88% (604) | 84% (584) | 90% (63) |
| *tetM* | 74% (1055) | 74% (131) | 58% (68) | 82% (341) | 68% (181) | 89% (195) | 71% (520) | 78% (535) | 71% (492) | 76% (53) |
| *tetO* | 13% (184) | 2% (4) | 25% (29) | 17% (69) | 18% (49) | 1% (2) | 14% (105) | 12% (79) | 14% (100) | 19% (13) |
| *tetL* | 1% (16) | 0 | 1% (1) | 3% (13) | 0 | 0 | 1% (6) | 1% (10) | 1% (5) | 0 |
|  |  |  |  |  |  |  |  |  |  |  |
| MLS_B_^b^ | 20% (281) | 30% (53) | 12% (14) | 19% (79) | 32% (85) | 14% (30) | 24% (179) | 15% (102) | 25% (172) | 26% (18) |
| *ermA* | 4% (53) | 11% (19) | 3% (3) | 0 | 12% (31) | 0 | 6% (41) | 2% (12) | 6% (40) | 0 |
| *ermB* | 12% (172) | 18% (32) | 9% (11) | 17% (71) | 15% (41) | 1% (2) | 14% (100) | 11% (72) | 14% (94) | 21% (15) |
| *ermT* | 0.4% (6) | 0 | 0 | 1% (3) | 1% (2) | 0.5% (1) | 1% (6) | 0 | 1% (6) | 0 |
| *lnuB* | 0.2% (3) | 0 | 1% (1) | 0.2% (1) | 0.4% (1) | 0 | 0 | 0.4% (3) | 0 | 0 |
| *lsaC* | 2% (26) | 0 | 0 | 0 | 7% (19) | 2% (5) | 2% (17) | 1% (9) | 2% (17) | 0 |
| *lsaE* | 0.2% (3) | 0 | 1% (1) | 0.2% (1) | 0.4% (1) | 0 | 0 | 0.4% (3) | 0 | 0 |
| *mefA/msrD* | 4% (62) | 1% (2) | 0 | 6% (24) | 4% (11) | 10% (22) | 5% (36) | 4% (26) | 5% (36) | 7% (5) |
|  |  |  |  |  |  |  |  |  |  |  |
| AMG^c^ | 6% (91) | 1% (1) | 5% (6) | 15% (64) | 4% (11) | 0.5% (1) | 7% (50) | 6% (41) | 7% (48) | 17% (12) |
| *aac(6')-aph(2'')* | 0.5% (7) | 0 | 0 | 0 | 1% (2) | 0.5% (1) | 1% (6) | 0.1% (1) | 1% (6) | 0 |
| *ant(6-Ia)* | 5% (76) | 0 | 5% (6) | 15% (64) | 2% (6) | 0 | 5% (36) | 6% (40) | 5% (35) | 17% (12) |
| *aph(3'-III)* | 6% (84) | 0 | 5% (6) | 15% (64) | 4% (10) | 0 | 6% (43) | 6% (41) | 6% (41) | 17% (12) |
| *aadE* | 5% (70) | 1% (1) | 4% (5) | 15% (64) | 0 | 0 | 4% (32) | 6% (38) | 4% (31) | 17% (12) |
|  |  |  |  |  |  |  |  |  |  |  |
| CHL^d^ | 1% (16) | 0 | 0 | 0 | 4% (12) | 0 | 2% (15) | 0.1% (1) | 2% (14) | 0 |
| *cat(pc194)* | 1% (9) | 0 | 0 | 0 | 2% (5) | 0 | 1% (8) | 0.1% (1) | 1% (7) | 0 |
| *catQ* | 0.5% (7) | 0 | 0 | 0 | 3% (7) | 0 | 1% (7) | 0 | 1% (7) | 0 |

^a^ Overall prevalence of tetracycline resistance genes

^b^ Overall prevalence of MLS_B_ resistance genes

^c^ Overall prevalence of aminoglycoside resistance genes

^d^ Overall prevalence of chloramphenicol resistance genes
